# Biogeographic patterns, assembly processes, and functional potential of root-associated microbiomes across the native range of the endangered orchid *Changnienia amoena*

**DOI:** 10.3389/fmicb.2026.1752368

**Published:** 2026-02-10

**Authors:** Wenhao Zhu, Zhanghua Quan, Ren Wang, Xingjian Liu, Jiayu Zhou

**Affiliations:** 1 Institute of Botany, Jiangsu Province and Chinese Academy of Sciences, Nanjing, China; 2 Nanjing University of Chinese Medicine, Nanjing, China; 3 College of Biological Sciences and Technology, Yili Normal University, Yining, China; 4 Jiangsu Key Laboratory for Conservation and Utilization of Plant Resources, Nanjing, China

**Keywords:** biogeographic patterns, *Changnienia amoena*, community assembly, microbial functions, orchid mycorrhizal fungi, root-associated microbiomes, soil nutrients

## Abstract

**Introduction:**

*Changnienia amoena* is an endangered orchid endemic to China, with notable medicinal and ornamental significance. Like other orchids, it relies heavily on microbial symbionts for germination, growth, and survival; however, its associated microbiomes remain poorly understood.

**Methods:**

This study employed high-throughput amplicon sequencing targeting bacterial 16S rRNA and fungal ITS regions and provided an in-depth characterization of the root-associated microbiomes of *C. amoena* across its native range.

**Results:**

Both bacterial and fungal communities exhibited significant biogeographic patterns, but only fungal Shannon-Wiener diversity showed a decreasing trend with increasing latitude. The neutral model revealed predominantly stochastic assembly of bacterial communities. In contrast, fungal communities exhibited a lower estimated migration rate, indicating a relatively stronger influence of non-neutral processes on their assembly. Soil nutrients emerged as key environmental drivers shaping microbial communities. Functional predictions highlighted multiple bacterial functions related to nitrogen cycling and the enrichment of mycorrhizal fungi, suggesting the functional potential of root-associated microbes in promoting plant nutrient acquisition, growth, and environmental adaptation. Co-occurrence network analysis further revealed positive associations between mycorrhizal fungi and potentially beneficial bacteria based on taxonomic inference.

**Discussion:**

This study reveals distinct biogeographic patterns, assembly processes, and functional potentials of root-associated microbiomes of *C. amoena*, which not only expands the understanding of the plant–soil-microbe interactions, but also provides crucial insights for manipulating them for the conservation of this endangered orchid.

## 1 Introduction

*Changnienia amoena* S. S. Chien is the sole representative of the monotypic genus *Changnienia*, and therefore occupies a unique and important position in taxonomy (Li and Ge, 2006). It is endemic to eastern and central China and narrowly distributed in the mountainous regions (Liu et al., 2025). *C. amoena* has long been used in traditional Chinese medicine for its antipyretic, antitussive, expectorant, and anti-inflammatory activities (Liu et al., 2024). However, its populations are declining rapidly due to habitat fragmentation and overexploitation. Consequently, this species is listed as a National Key Protected Wild Plant in China and classified as endangered on the International Union for Conservation of Nature (IUCN) Red List. For terrestrial orchids, the establishment of close interactions with orchid mycorrhizal fungi (OMF) is essential for seed germination, protocorm development, and seedling establishment, further influencing plant population dynamics and spatial distribution (McCormick et al., 2018). It is noteworthy that belowground interactions between orchids and their microbiomes are far more complex (Waud et al., 2017). Other fungal taxa in the root endosphere and rhizosphere (here collectively referred to as root-associated compartments) can also enhance nutrient acquirement and plant growth (Toju et al., 2013). Moreover, root-associated bacteria (here referring to bacteria inhabiting the root endosphere and rhizosphere) can contribute to orchid health and environmental adaptation and be classified into plant growth-promoting bacteria, mycorrhization helper bacteria (MHBs), and orchid holobiont constituents (Kaur and Sharma, 2021). Collectively, the establishment, distribution, and long-term persistence of endangered orchids are significantly affected by root-associated microbiomes.

Habitat fragmentation is not only a major driver of plant endangerment (Krauss et al., 2010), but also leads to the spatial distribution and environment-specific evolution of plant root-associated microbiomes (Kraemer and Boynton, 2017). Root-associated microbiomes are critical components of plant microecosystem, contributing to nutrient acquisition, stress tolerance, pathogen resistance, and overall plant fitness (Trivedi et al., 2020). Therefore, elucidating their biogeographic patterns, assembly processes, and functional potential can provide new insights for the conservation of endangered orchids and broaden conservation concepts from protecting the plants alone to maintaining a healthy and functional root-associated microecosystem. Microbial community displays biogeographic patterns in composition, diversity, and functions, shaped by both stochastic processes (e.g., dispersal limitation) and deterministic factors (e.g., environmental filtering) (Hanson et al., 2012). Among the latter, soil physicochemical properties, particularly nutrient availability, are recognized as key factors driving microbial community assembly (Xu et al., 2022). As root-associated microbes are primarily recruited from the surrounding soil (Bulgarelli et al., 2013; Urbina et al., 2018), variation in soil properties across habitats is expected to shape their assembly. However, the biogeographic patterns and assembly processes of root-associated microbiomes, particularly in endangered plant species, are still relatively underexplored.

Given its taxonomic distinctiveness and medicinal importance, *C. amoena* receives increasing attention in recent years. However, previous studies have focused mainly on its distribution, ecology, and conservation biology (Li and Ge, 2006; Liu et al., 2024; Liu et al., 2025). Its root-associated microbiomes remain largely uncharacterized. A previous culture-based study isolated 18 fungal taxa from its roots (Jiang et al., 2011). However, most microbes cannot be cultivated (Liu et al., 2022). As a monotypic lineage, the unique evolutionary history of *C. amoena* may be associated with distinctive host traits that potentially influence the assembly of its root-associated microbiomes (Bouffaud et al., 2014; Schroeder et al., 2019). Therefore, the diversity, composition, and ecological functions of the root-associated microbiomes of *C. amoena* need further investigation. We hypothesize that root-associated bacterial and fungal communities of *C. amoena* exhibit distinct biogeographic patterns and assembly mechanisms across its native geographic distribution range, and that different microbial taxa possess diverse functional potentials that may influence the survival and growth of this endangered orchid. In this study, we systematically investigated the root-associated bacterial and fungal communities of *C. amoena* across six native populations distributed across the geographic range of this species. High-throughput amplicon sequencing targeting bacterial 16S rRNA and fungal ITS regions was employed to assess microbial diversity, composition, and spatial distribution. We further examined the influences of soil nutrients on microbial community assembly and performed functional prediction and co-occurrence network construction to infer the ecological roles of root-associated microbiomes of *C. amoena*. Our findings provide novel insights into plant–soil-microbe interactions in an endangered orchid and offer microecological insights to support its conservation.

## 2 Materials and methods

### 2.1 Plant and soil sampling

*Changnienia amoena* typically flowers in April, which is a key phenological stage for plant-microbe interactions (Chaparro et al., 2014). Therefore, in April 2023, a total of 32 individuals of *C. amoena* were collected from 6 major native habitats across China: 6 individuals of *C. amoena* from Kang County, Longnan City, Gansu Province (LongNan, 105°43′E; 33°18’N); 6 from Jigongshan National Nature Reserve, Xinyang City, Henan Province (XinYang, 114°2′E; 31°47’N); 6 from Xuan’en County, Enshi Tujia and Miao Autonomous Prefecture, Hubei Province (EnShi, 109°49′E; 29°57’N); 5 from Shennongjia Forestry District, Hubei Province (ShenNongJia, 110°22′E; 31°26’N); 3 from Gaowangjie National Nature Reserve, Guzhang County, Xiangxi Tujia and Miao Autonomous Prefecture, Hunan Province (XiangXi, 110°4′E; 28°37’N); and 6 from Tongjiang County, Bazhong City, Sichuan Province (BaZhong, 107°11′E; 31°56’N). During the field sampling, no visible disease symptom was observed on *C. amoena* individuals, and all sampled plants were healthy. At the same time, bulk soil samples were collected from each habitat, including 6 from LongNan, 6 from XinYang, 6 from EnShi, 5 from ShenNongJia, 3 from XiangXi and 6 from BaZhong. Sampling sites within each habitat were spaced at least five meters apart. Soil samples were air-dried, ground, and passed through a 2-mm sieve prior to analysis.

Rhizosphere soil was sampled according to Attia et al. (2022) with some modifications. In detail, loose soil attached to the roots were gently brushed off using a clean brush, leaving an approximately 1 mm thick layer of soil tightly attached to roots. The roots were then immersed in 25 mL of Phosphate Buffered Saline (PBS) buffer in a sterile tube and then vortexed vigorously for 15 s to obtain microbes in the thin layer of soil, representing the rhizosphere fraction. The same roots from previous step were transferred in a new tube containing 25 mL of PBS buffer and subjected to an ultrasonic water bath to further detached microbes on the root surface, representing the rhizoplane fraction. Sonication was performed for 30 s at 70 Hz followed by a pause for 30 s, repeated for 10 cycles. The entire process was carried out at 4 °C to prevent overheating. The suspensions obtained from both steps were intentionally combined and centrifuged at 12,000 *g* for 10 min at 4 °C. The resulting pellet was collected as the rhizosphere soil sample, while the remaining roots were collected as the root sample. Both root endosphere and rhizosphere samples were flash-frozen in liquid nitrogen and stored at −80 °C until DNA extraction.

### 2.2 Soil nutrient analysis

Soil total nitrogen (TN) was determined using the Kjeldahl digestion method, total phosphorus (TP) was measured by the molybdenum antimony colorimetric method after fusion with NaOH, while total potassium (TK) was quantified by flame photometry after fusion with NaOH (Jiang et al., 2024). Soil available nitrogen (AN) was determined using the alkaline diffusion method, available phosphorus (AP) was measured by the molybdenum-antimony colorimetric assay after extraction with NaHCO_3_, available potassium (AK) was quantified by flame photometry after extraction with NH_4_OAc, while soil organic carbon (SOC) was measured using the K_2_Cr_2_O_7_ oxidation–reduction titration method (Wang et al., 2024). Differences in the TN, AN, TP, AP, TK, AK, and SOC of soil collected from different locations were statistically evaluated using One-Way Analysis of Variance (ANOVA).

### 2.3 DNA extraction and amplicon sequencing

The total DNA was extracted from root and rhizosphere samples using the E. Z. N. A.^®^ Plant DNA Mini Kit (OMEGA Bio-tek Inc., Norcross, GA, United States) and the FastDNA™ SPIN Kit for Soil (MP Biomedicals, Solon, OH, United States) according to the manufacturer’s instructions, respectively. The quality of DNA was controlled by agarose gel electrophoresis, and the concentration of DNA was quantified using a NanoDrop™ 1,000 Spectrophotometer (NanoDrop Technologies, Wilmington, DE, United States). DNA was stored at −80 °C until subsequent sequencing.

The concentration of DNA in all samples was normalized to 10 ng/μL. The primers 799F (5’-AACMGGATTAGATACCCKG-3′) and 1223R (5′-CCATTGTAGTACGTGTGTA-3′) were used to amplify the V5-V7 hypervariable region of the bacterial 16S rRNA gene (Biget et al., 2024). The primers ITS86F (5’-GTGAATCATCGAATCTTTGAA-3′) and ITS4 (5’-TCCTCCGCTTATTGATATGC-3′) were used to amplify the fungal internal transcribed spacer (ITS) 2 region (Jacquemyn et al., 2014). Each Polymerase Chain Reaction (PCR) was performed in 10 μL mixture containing 0.2 μL TransStart^®^ TopTaq DNA Polymerase (TransGen Biotech Co., Ltd., Beijing, China), 0.2 μM of each primer (10 μM), and 1 μL template DNA. The thermal cycling program consisted of an initial denaturation at 94 °C for 2 min, followed by 27 cycles of denaturation at 94 °C for 30 s, annealing at 55 °C for 30 s, and extension at 72 °C for 1 min, with a final extension at 72 °C for 10 min. PCR products showing a distinct band of approximately 400 bp for bacteria and 450 bp for fungi were visualized by agarose gel electrophoresis, excised from the gel, and purified using AxyPrep™ DNA Gel Extraction Kit (Corning Inc., Glendale, AZ, United States). Products from three independent PCR replicates per sample were pooled in equal volumes to minimize amplification bias. Amplicons of the expected size were sequenced on the Illumina NovaSeq 6,000 platform (Illumina, Inc., San Diego, CA, United States) to generate paired-end reads of approximately 250 bp. A standard bacterial/fungal genomic DNA mix was used as the positive control, and sterile water was as the negative control.

Raw sequencing reads were processed using QIIME 2 (Bolyen et al., 2019). Adapter and primer sequences were trimmed using the cutadapt plugin. Quality control and amplicon sequence variant (ASV) identification were performed using the DADA2 plugin (Callahan et al., 2016). Rarefaction curves of both bacterial and fungal communities approached saturation with increasing sequencing depth (Supplementary Figure S1), indicating that the sequencing effort was sufficient to capture the majority of microbial diversity. Taxonomic assignments of bacterial ASV representative sequences were conducted using a pre-trained Naive Bayes classifier based on the SILVA database (version 138.1) with a confidence threshold of 0.8, while taxonomic assignments of fungal ASV representative sequences were conducted using a pre-trained Naive Bayes classifier based on the UNITE database (version 9.0) with a confidence threshold of 0.6. The raw 16S rRNA and ITS amplicon sequencing data have been deposited in the NCBI Sequence Read Archive (SRA) under accession numbers PRJNA1322054 and PRJNA1321960, respectively.

### 2.4 Data analysis

Downstream data analyses were conducted in R (version 4.5.0). To minimize biases arising from unequal sequencing depths, bacterial and fungal ASV tables were rarefied to the lowest sequencing depth among all samples, which was 59,289 reads for bacteria (Supplementary Table S1) and 51,102 reads for fungi (Supplementary Table S2). Alpha diversity indices were calculated using the vegan package (Oksanen et al., 2007). Differences in Shannon-Wiener diversity index across sampling locations and root-associated compartments were statistically evaluated using Two-Way ANOVA, respectively. Bray–Curtis dissimilarity matrices were computed using the vegdist function, and principal coordinates analysis (PCoA) was performed using the cmdscale function, both from the vegan package. Permutational multivariate analysis of variance (PERMANOVA) was employed to assess the individual effect of location and compartment on microbial communities (permutation number = 999). The top 20 most abundant bacterial and fungal genera were identified based on their average relative abundance across all samples. Stacked bar plots were created using the tax_stackplot function in the ggplot2 package to visualize taxonomic composition (Liu et al., 2021). Core Amplicon Sequence Variants (ASVs) were identified following the framework proposed by Qiao et al. (2024). Briefly, Venn diagrams were drawn to determined bacterial and fungal ASVs across locations with each compartment. Ecological niche breadth was then calculated for individual ASVs within the same location and compartment, and the top 10% bacterial or fungal ASVs were classified as generalists. ASVs that were both shared across locations and classified as generalists were defined as core microbiome members.

To explore the influences of soil nutrients on bacterial and fungal communities, several statistical analyses were performed (Zhou et al., 2024). Firstly, the ASV count was subjected to Hellinger transformation to reduce the impact of rare taxa, while the soil variables, including TN, AN, TP, AP, TK, AK, and SOC, were normalized by log1p-transformation. Canonical correspondence analysis (CCA) was performed to test the relationship between bacterial/fungal ASV matrix and soil nutrients. Generalized additive model fitting was incorporated in the PCoA ordination (Bray–Curtis distance) to assess the relationship between the root endosphere/rhizosphere bacterial/fungal community composition and each individual soil nutrient using the ordisurf function in the vegan package. The species richness of bacterial and fungal communities in each location was calculated using the specnumber function in the vegan package. Next, the linear models were fitted using the lm function in the stats package to show the relationship between bacterial / fungal richness and each individual soil nutrient.

Microbial community assembly processes were evaluated using a neutral model, implemented via the neutral.fit function (Sloan et al., 2006). Partial least squares path modeling (PLS-PM) was conducted using the plspm package to disentangle the relationships among geographic location, soil nutrient, and root endosphere/rhizosphere bacterial/fungal community (Tenenhaus et al., 2005). The first principal coordinate axis (PCoA1), derived from community dissimilarity matrices, was used to represent bacterial and fungal community structure in each compartment. Path coefficients (*β*) represent standardized direct effects among latent variables, while *R*^2^ values indicate the proportion of variance explained in endogenous variables. The functions of bacterial community were predicted based on Functional Annotation of Prokaryotic Taxa (FAPROTAX) database (Louca et al., 2016), while the ecological guilds of fungal community were assigned based on FUNGuild database (Nguyen et al., 2016). Co-occurrence networks were constructed by jointly analyzing bacterial and fungal ASVs based on root endosphere and rhizosphere samples collected from the same native habitat. Prior to network construction, only ASVs present in at least 20% of samples were retained. Among these, ASVs with a total relative abundance greater than 0.01% across all samples were selected for Spearman correlation analysis. Only strong and significant correlations (Spearman’s *ρ* ≥ 0.7, *p* < 0.0001) were retained as robust associations. To reduce false positives, *p* values were adjusted for multiple testing using the Benjamini–Hochberg procedure prior to network construction (Li et al., 2023).

## 3 Results

### 3.1 Microbial diversity in root endosphere and rhizosphere of *C. amoena* across native habitats

**Figure 1:**
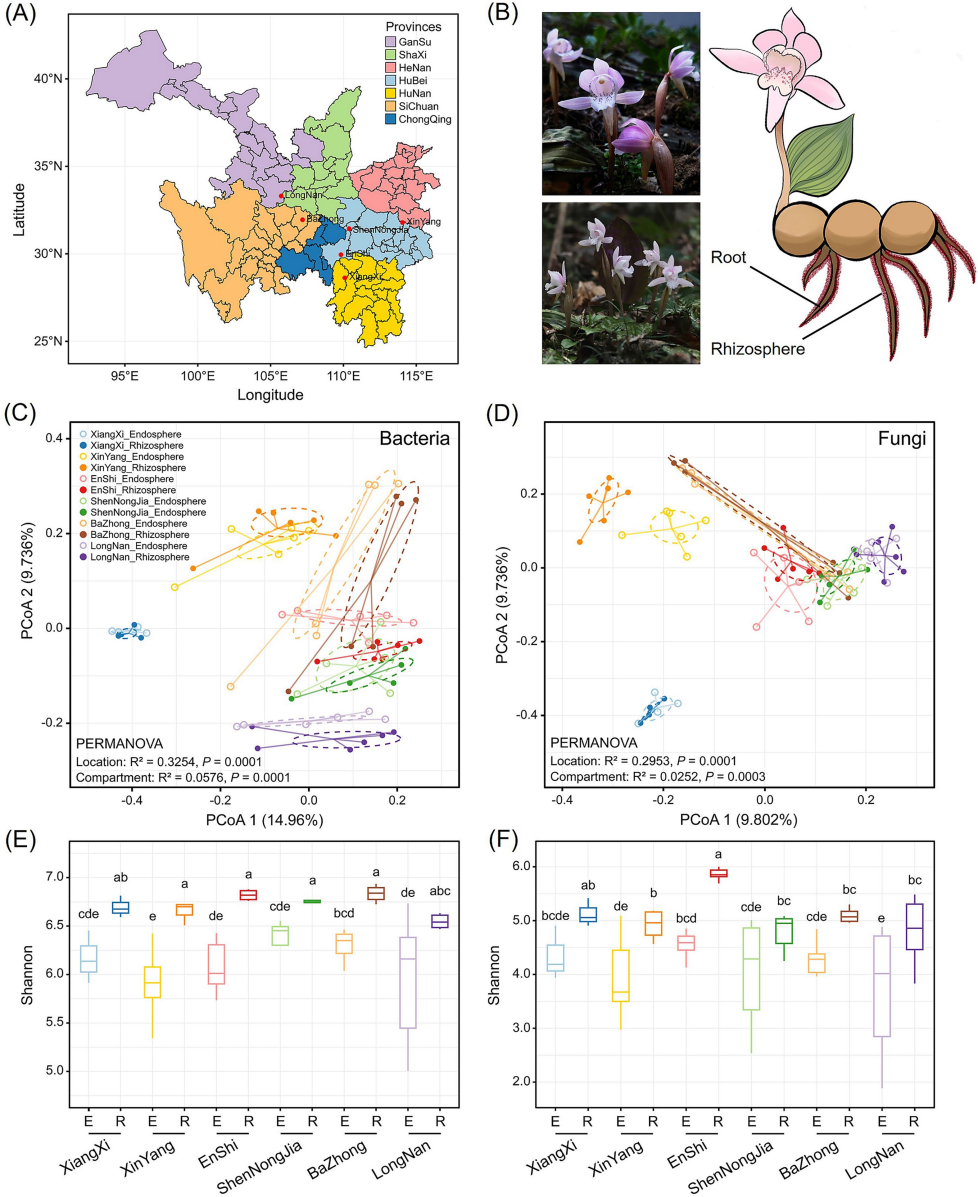
Diversity analysis of bacterial and fungal communities in the root endosphere and rhizosphere of *Changnienia amoena* across native habitats. **(A)** A map of sampling locations in the eastern and central regions of China. **(B)** *C. amoena* individuals in their native habitats and a schematic diagram illustrating the sample compartments (Root and Rhizosphere). **(C,D)** Principal coordinates analysis (PCoA) of bacterial and fungal communities based on Bray–Curtis dissimilarities. Statistical significance of community differences among locations or compartments was assessed using permutational multivariate analysis of variance (PERMANOVA). **(E,F)** Boxplots of Shannon–Wiener diversity index for bacterial **(E)** and fungal **(F)** communities in the root endosphere (E) and rhizosphere (R) across habitats. Horizontal lines within boxes indicate medians. Tops and bottoms of boxes indicate 75th and 25th quartiles, respectively. Upper and lower whiskers extend 1.5× the interquartile range from the upper edge and lower edge of the box, respectively. Different letters indicate significant differences among different locations and compartments (*p* < 0.05) based on two-way ANOVA. The number of samples (*n*) per habitat is as follows: LongNan (*n* = 6), XinYang (*n* = 6), EnShi (*n* = 6), ShenNongJia (*n* = 5), XiangXi (*n* = 3), and BaZhong (*n* = 6).

Plant sampling was conducted in six native habitats of *C. amoena* (Figure 1A). For each location, the root and rhizosphere soil samples were collected (Figure 1B). Then, the amplicon sequencing was performed to reveal the bacterial and fungal community composition and diversity. The composition of root-associated bacterial communities varied significantly across geographic locations (*R*^2^ = 0.3254, *p* = 0.0001) and root-associated compartments (*R*^2^ = 0.0576, *p* = 0.0001), with location having a stronger influence. Bacterial communities from XiangXi were clearly distinct from those at other locations, while those from EnShi, ShenNongJia, and BaZhong were highly similar to each other (Figure 1C). A similar pattern was observed for fungal communities (Figure 1D), with location having a stronger influence (*R*^2^ = 0.2953, *p* = 0.0001) compared to compartment (*R*^2^ = 0.00252, *p* = 0.0003). Fungal communities from XiangXi were clearly distinct, while those from EnShi, ShenNongJia, BaZhong, and LongNan were highly similar. Further analysis revealed that the rhizosphere of *C. amoena* harbored more diverse bacterial (Figure 1E) and fungal communities compared to the root endosphere (Figure 1F). The Shannon diversity indices of bacterial communities did not show significant difference among locations in either the root endosphere or rhizosphere. Similarly, the Shannon indices of fungal communities in the root endosphere did not differ significantly across locations; however, that in the rhizosphere from EnShi was higher than those observed at other locations.

### 3.2 Microbial composition and functional prediction in root endosphere and rhizosphere of *C. amoena* across native habitats

**Figure 2:**
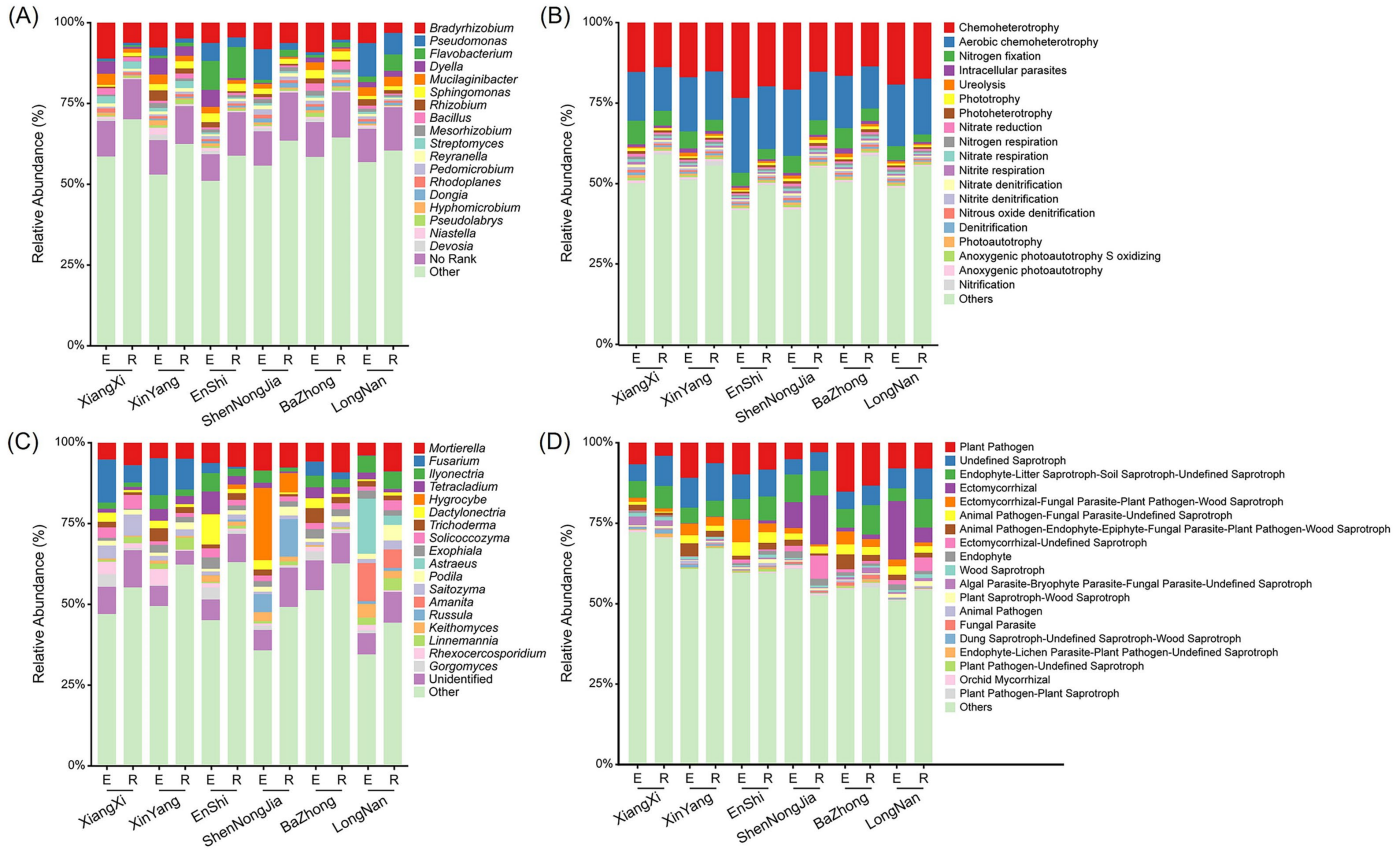
Taxonomic composition and functional potential of bacterial and fungal communities in the root endosphere (E) and rhizosphere (R) of *Changnienia amoena* across different habitats. **(A,C)** Relative abundance of bacterial **(A)** and fungal **(C)** taxa at genus level. The top 20 most abundant genera are shown. **(B,D)** Predicted ecological functions of bacterial communities using FAPROTAX **(B)** and predicted trophic and ecological guilds of fungal communities using FUNGuild **(D)**. The top 20 most abundant functions are shown. The number of samples (*n*) per habitat is as follows: Longnan (*n* = 6), Xinyang (*n* = 6), Enshi (*n* = 6), Shennongjia (*n* = 5), Xiangxi (*n* = 3), and Bazhong (*n* = 6).

The dominant bacterial genera associated with the roots of *C. amoena* were relatively consistent across locations (Figure 2A). The top 10 most abundant ones included *Bradyrhizobium*, *Pseudomonas*, *Flavobacterium*, *Dyella*, *Mucilaginibacter*, *Sphingomonas*, *Rhizobium*, *Bacillus*, *Mesorhizobium*, and *Streptomyces*. In detail, the relative abundance of *Bradyrhizobium* was higher in the endosphere than in the rhizosphere. *Pseudomonas* was more abundant in the endosphere of *C. amoena* from ShenNongJia and LongNan, while *Flavobacterium* was more abundant in both compartments from EnShi. Notably, *Bradyrhizobium*, *Rhizobium*, and *Mesorhizobium* were consistently present in all the samples, which may be associated with mycorrhizal symbiosis in *C. amoena*. Similarly, the dominant fungal genera associated with the roots of *C. amoena* were also relatively consistent across locations (Figure 2C). The top 10 most abundant ones included *Mortierella*, *Fusarium*, *Ilyonectria*, *Tetracladium*, *Hygrocybe*, *Dactylonectria*, *Trichoderma*, *Solicoccozyma*, *Exophiala*, and *Astraeus*. The relative abundances of most genera were higher in the endosphere than in the rhizosphere. Notably, *Hygrocybe* and *Russula* were most abundant in both compartments of *C. amoena* from ShenNongJia, while *Astraeus* and *Amanita* were most abundant in those from LongNan. More importantly, *Astraeus*, *Amanita* and *Russula* are frequently reported as mycorrhizal fungi and are likely to play essential roles in the growth and development of *C. amoena*.

Furthermore, a total of 28 bacterial functional groups and 27 fungal guilds were predicted using FAPROTAX and FUNGuild, respectively. The top 20 most abundant bacterial functional potential were similar in both root endosphere and rhizosphere across habitats (Figure 2B). Notably, several predicted bacterial functional potentials were involved in nitrogen cycling processes, including nitrogen fixation, nitrate reduction, nitrogen respiration, nitrate respiration, nitrite respiration, nitrate denitrification, nitrite denitrification, nitrous oxide denitrification, denitrification, and nitrification. These functional potentials indicated that bacterial communities might play key roles in both nitrogen input and transformation in the root-associated compartments of *C. amoena*. Among these, nitrogen fixation was the most dominant predicted nitrogen-related function, suggesting a potential enhancement of nitrogen acquisition by host plants, which was consistent with the enrichment of *Bradyrhizobium*, *Rhizobium*, and *Mesorhizobium* (Figure 2A). On the other hand, the ecological guild prediction of fungal communities showed a higher relative abundance of ectomycorrhizal and orchid mycorrhizal trophic modes, particularly in samples from ShenNongJia and LongNan (Figure 2D). This pattern was consistent with the higher relative abundances of ectomycorrhizal genera such as *Astraeus*, *Amanita*, and *Russula* in these locations (Figure 2C). Additionally, saprotrophic fungi were also abundant in the root-associated compartments across habitats, likely contributing to organic matter decomposition and nutrient cycling. These findings suggested that the root-associated fungal communities might facilitate nutrient acquisition and improve the growth and adaptation of host plants.

### 3.3 Core microbiome members in root-associated compartments of *C. amoena* across native habitats

In this study, bacterial and fungal ASVs that were both shared among habitats and exhibited generalist ecological characteristics in the root endosphere and rhizosphere were defined as core microbiome members. Venn diagrams revealed that 345 bacterial ASVs (Supplementary Figure S2A) and 38 fungal ASVs were consistently shared across all habitats in the root endosphere (Supplementary Figure S2C), while 403 bacterial ASVs (Supplementary Figure S2B) and 62 fungal ASVs were shared across habitats in the rhizosphere (Supplementary Figure S2D). Ecological niche breadth analysis further identified a large proportion of ASVs as generalists, including 4,061 bacterial (Supplementary Figure S2A) and 2,102 fungal ASVs as generalists in the root endosphere (Supplementary Figure S2C), and 4,054 bacterial (Supplementary Figure S2B) and 2,102 fungal ASVs as generalists in the rhizosphere (Supplementary Figure S2D). Based on their occurrence across habitats and generalist ecological characteristics, 345 bacterial (Supplementary Figure S2A) and 37 fungal ASVs in the root endosphere (Supplementary Figure S2C), as well as 403 bacterial (Supplementary Figure S2B) and 62 fungal ASVs in the rhizosphere (Supplementary Figure S2D), were defined as core microbiome members.

**Figure 3:**
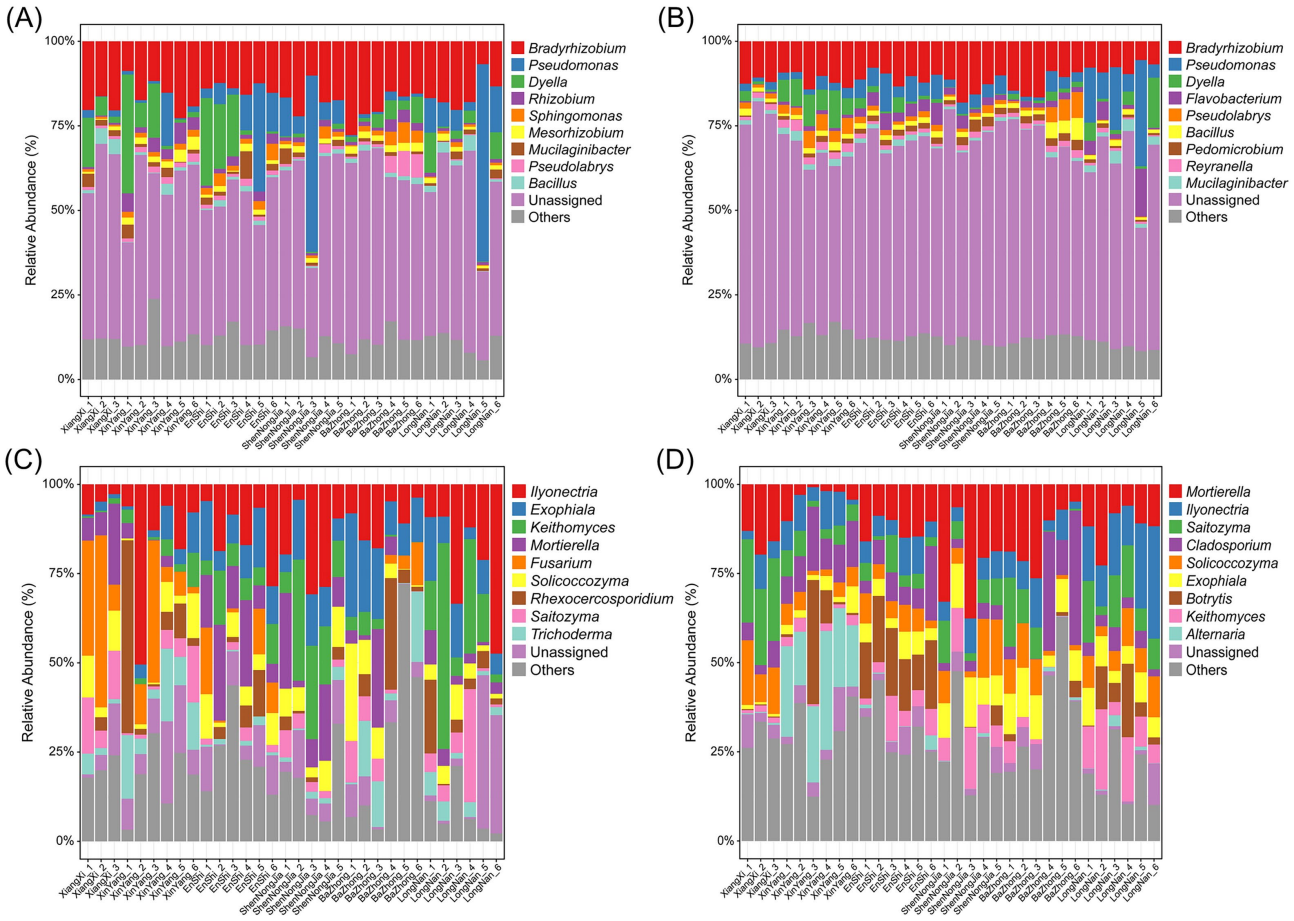
Taxonomic composition of bacterial and fungal core amplicon sequence variants (ASVs) in the root endosphere and rhizosphere of *Changnienia amoena* across different habitats. **(A,C)** Relative abundances of bacterial **(A)** and fungal **(C)** core ASVs at genus level in the root endosphere. **(B,D)** Relative abundances of bacterial **(B)** and fungal **(D)** core ASVs at genus level in the rhizosphere. The top 10 most abundant genera are shown. The number of samples (*n*) per habitat is as follows: Longnan (*n* = 6), Xinyang (*n* = 6), Enshi (*n* = 6), Shennongjia (*n* = 5), Xiangxi (*n* = 3), and Bazhong (*n* = 6).

Taxonomic classification showed that bacterial core ASVs in root-associated compartments were dominated by several genera commonly associated with plant-microbe interactions. In the root endosphere, bacterial core members were mainly affiliated with *Bradyrhizobium*, *Pseudomonas*, *Dyella*, *Rhizobium*, and *Sphingomonas* (Figure 3A), whereas the rhizosphere core bacterial community exhibited a similar taxonomic composition, with *Bradyrhizobium*, *Pseudomonas*, *Dyella*, *Flavobacterium*, and *Pseudolabrys* as dominant genera (Figure 3B). Fungal core ASVs displayed more compartment-specific taxonomic patterns. In the root endosphere, *Ilyonectria*, *Exophiala*, *Keithomyces*, *Mortierella*, and *Fusarium* were dominant (Figure 3C), whereas *Mortierella*, *Ilyonectria*, *Saitozyma*, *Cladosporium*, and *Solicoccozyma* were dominant genera in the rhizosphere (Figure 3D). Overall, both the number and taxonomic composition of core microbial members differed between the root endosphere and rhizosphere, indicating compartment-specific patterns of core microbiome assembly.

### 3.4 Microbial co-occurrence patterns in root-associated compartments of *C. amoena* across native habitats

**Figure 4:**
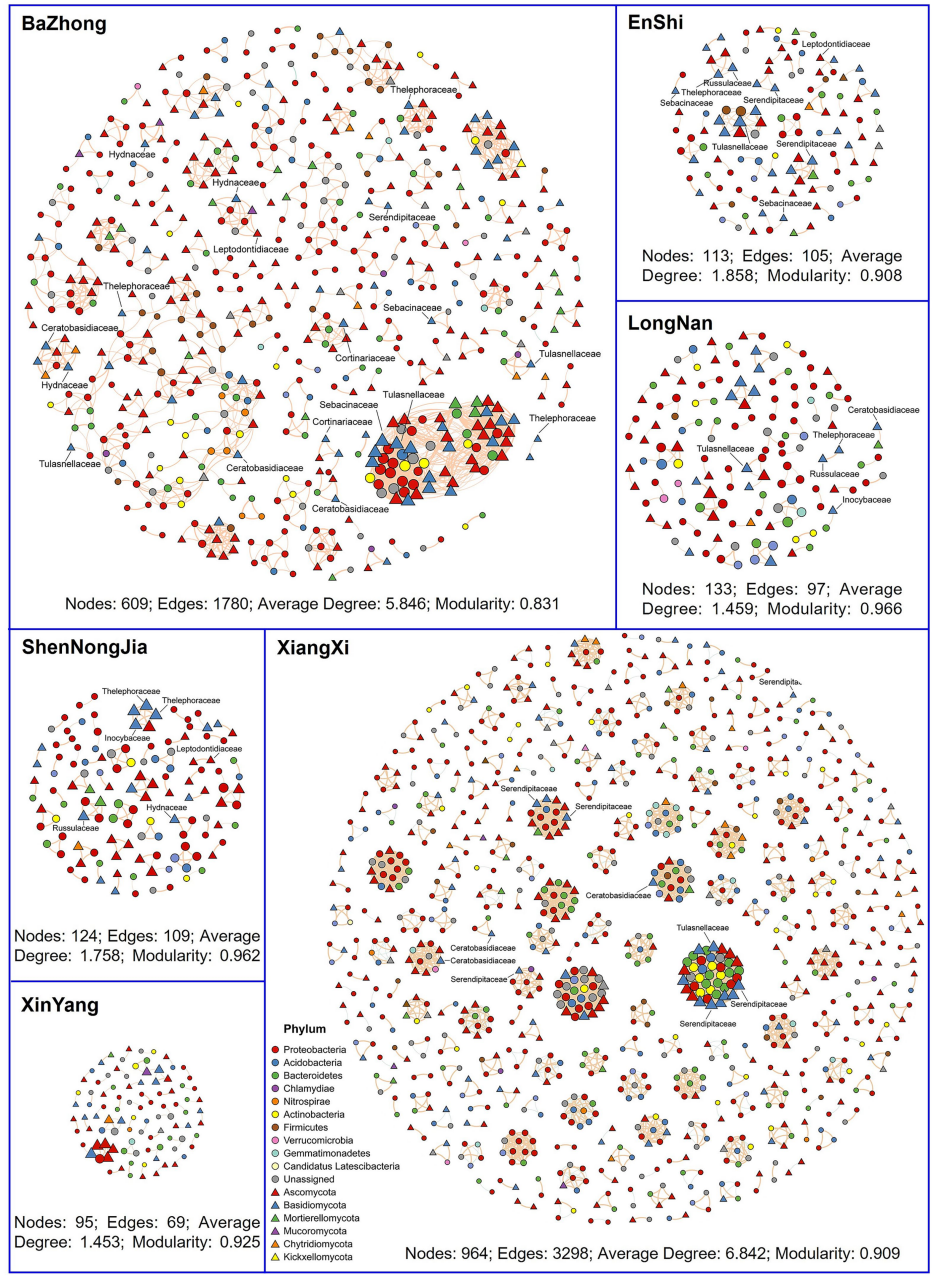
Overall co-occurrence networks of bacterial and fungal communities constructed based on the pairwise Spearman’s correlations between amplicon sequence variants (ASVs). Edges represent strong (Spearman *ρ* ≥ 0.7 or *ρ* ≤ −0.7) and significant (*p* < 0.0001) correlations. Nodes are colored according to different phyla. Circles denote bacterial ASVs, while triangles denote fungal ASVs. In each panel, node size is proportional to the relative abundance of corresponding ASV. Pink and green edges indicate positive and negative correlations, respectively.

Network analysis revealed distinct microbial co-occurrence patterns across six native habitats of *C. amoena* (Figure 4). The root-associated microbial communities in XiangXi formed a highly connected network with a larger size (nodes = 964), greater connectivity (edges = 3,298), and higher complexity (average degree = 6.842), whereas the network in XinYang exhibited the weakest connectivity, characterized by fewer nodes (nodes = 95) and edges (edges = 69), and lower complexity (average degree = 1.453). However, all the microbial networks exhibited high modularity values (ranging from 0.831 to 0.966), with the highest observed in LongNan, indicating a high degree of community compartmentalization. Obviously, positive correlations dominated all the networks, reflecting prevalent cooperative interactions among root-associated microbes of *C. amoena*. However, some negative correlations existed in Bazhong and XiangXi, suggesting the potential microbial competition or antagonism. It should be noted that the co-occurrence networks were inferred from Spearman correlations, which reflect statistical associations rather than direct ecological interactions; thus, potential indirect or compositional effects could not be completely excluded.

Bacterial ASVs belonging to Proteobacteria, Bacteroidetes, and Actinobacteria consistently forming large and well-connected hubs with fungal ASVs, which were particularly observed in Bazhong and XiangXi. These fungal ASVs were dominated by Basidiomycota and Ascomycota. In contrast, less abundant fungal phyla such as Mortierellomycota and Chytridiomycota appeared more scattered and less interconnected in the networks. Notably, ASVs affiliated with mycorrhizal fungi frequently occupied central positions in the co-occurrence networks across all habitats except XinYang. These ASVs were assigned to Ceratobasidiaceae, Cortinariaceae, Hydnaceae, Inocybaceae, Leptodontidiaceae, Russulaceae, Sebacinaceae, Serendipitaceae, Thelephoraceae, and Tulasnellaceae. Interestingly, these mycorrhizal fungal ASVs exhibited significant positive relationships with numerous bacterial ASVs, many of which were affiliated with *Bradyrhizobium*, *Rhizobium*, *Mesorhizobium*, and *Nitrospira*. These findings suggested that interactions between mycorrhizal fungi and bacteria in the root-associated compartments of *C. amoena* might play essential roles in facilitating nutrient acquisition by host plants.

### 3.5 Assembly processes and biogeographic patterns of root-associated microbial communities of *C. amoena* across native habitats

**Figure 5:**
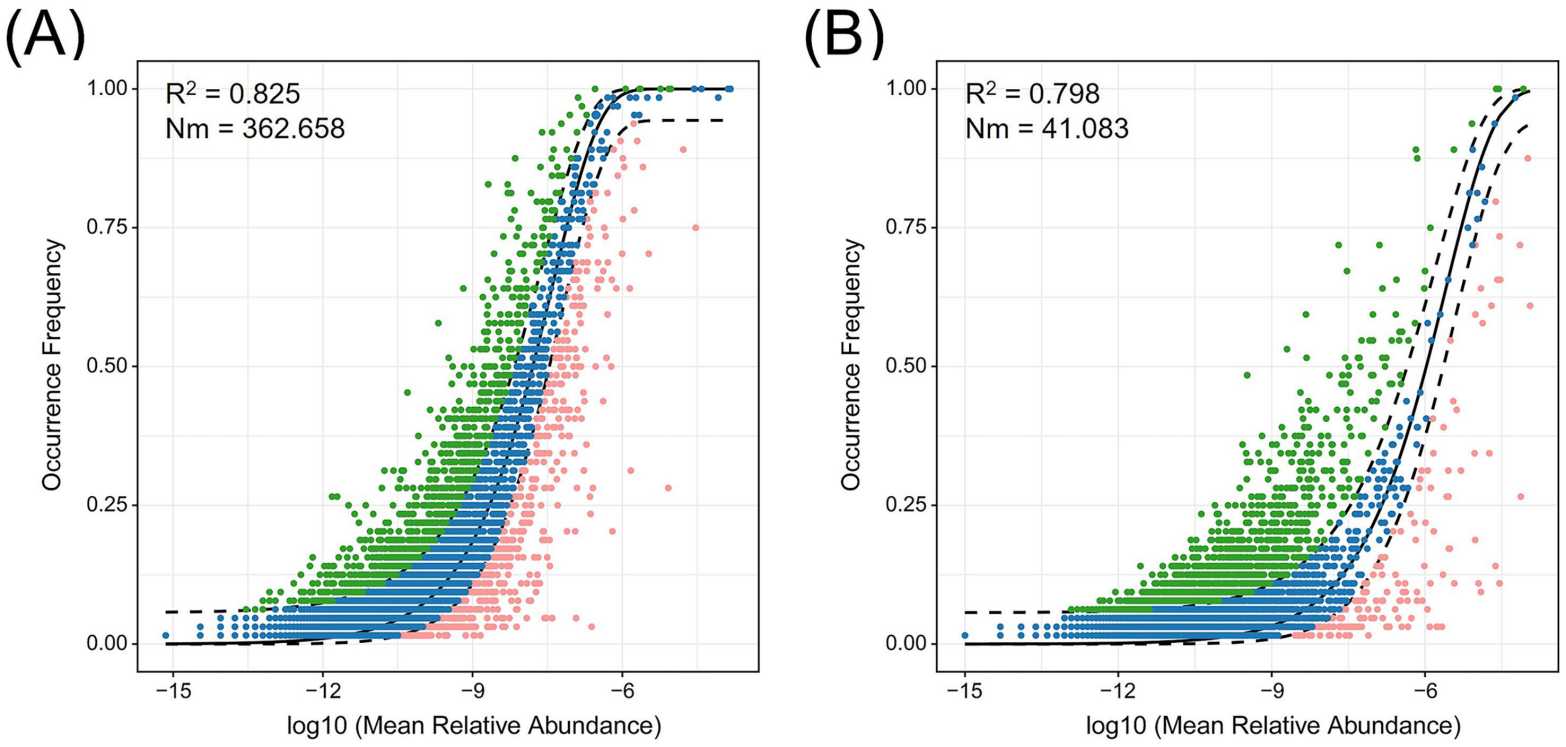
Fitting of neutral community model of bacterial **(A)** and fungal **(B)** community assembly. Amplicon sequence variants (ASVs) that occur more frequently than predicted by the model are shown in green while those that occur less frequently are shown in pink. The solid black line indicates the best fit to the model and the dashed black lines represent 95% confidence intervals around the model prediction. *Nm* indicates the metacommunity size times immigration, while *R*^2^ indicates the fit to this model.

The assembly processes of root-associated bacterial and fungal communities of *C. amoena* were evaluated using the neutral community model. Generally, the model provided a good fit for both microbial communities, explaining 82.5% of the variation in bacterial communities (Figure 5A) and 79.8% in fungal communities (Figure 5B). However, stochastic processes exert a stronger influence on the assembly of bacterial communities than on fungal communities. Additionally, the estimated migration rate (*Nm*) was substantially higher for bacterial community assembly (*Nm* = 362.658) than for fungal community assembly (*Nm* = 41.083). All these results indicated that the root-associated fungal communities exhibited more limited dispersal than bacterial communities, implying that the fungal community assembly was more strongly governed by spatial constraints and environmental factors.

**Figure 6:**
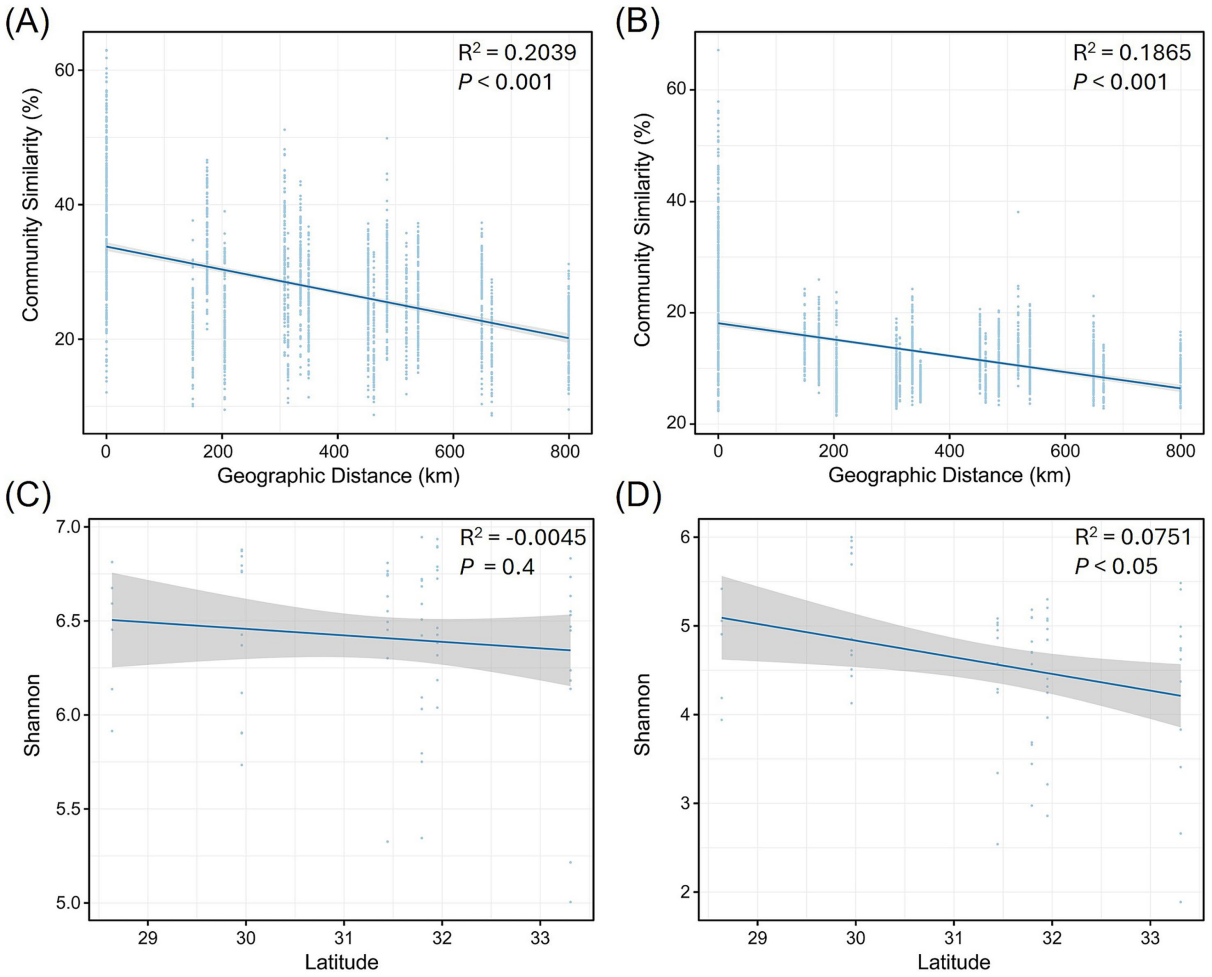
Correlations between geographic factors and root-associated microbial communities of *Changnienia amoena*. **(A,B)** Correlations between geographic distances and bacterial **(A)** and fungal community similarity **(B)**. **(C,D)** Correlations between latitude and bacterial **(C)** and fungal diversity **(D)**.

To investigate the biogeographic patterns of root-associated microbial communities of *C. amoena*, we first performed univariate linear regression analysis to assess the correlation between microbial community similarity and geographic distance among habitats. A significant negative correlation was observed for bacterial communities (*R*^2^ = 0.2039, *p* < 0.001), indicating that the similarity of bacterial communities significantly decreased with increasing geographic distances (Figure 6A). Although the correlation for fungal communities was slightly lower (*R*^2^ = 0.1865, *p* < 0.001), a significant decline in similarity was also detected as geographic distance increased (Figure 6B). Additionally, the correlations between microbial Shannon-Wiener diversity and the latitude of sampling locations were analyzed. The diversity of bacterial communities exhibited weak correlations with latitude (Figure 6C). In contrast, fungal diversity significantly decreased with increasing latitude (Figure 6D). These results indicated that geographic factors influence both bacterial and fungal community similarity, whereas fungal community diversity shows a stronger association with geographic gradients than bacterial diversity.

### 3.6 Soil nutrients driving root-associated microbial communities of *C. amoena* across native habitats

Since geographic locations had pronounced influences on root-associated microbial communities of *C. amoena* (Figure 6), we speculated that soil nutrient availability could shape microbial communities across habitats. Therefore, TN, AN, TP, AP, TK, AK, and SOC in soil collected from different native habitats were measured. The trends of TN and AN were consistent across habitats, with the highest levels observed in EnShi and the lowest in XiangXi and BaZhong (Supplementary Figures S3A,B). However, TP and AP exhibited divergent patterns. TP was highest in EnShi and lowest in XiangXi and LongNan, while AP peaked in BaZhong (Supplementary Figures S3C,D). TK and AK showed similar trends, with the highest level observed in ShenNongJia. However, TK was lowest in EnShi and LongNan, while AK was lowest in BaZhong (Supplementary Figures S3E,F). Moreover, SOC was highest in EnShi and lowest in XiangXi (Supplementary Figure S3G). Correlation analysis revealed strong associations among TN, AN, and SOC, with coefficients greater than 0.95 (Supplementary Figure S3H). These strong correlations might be attributed to the role of SOC as an energy source that stimulated microbial activities involved in N cycling, thereby enhancing the decomposition and mineralization of organic nitrogen (Shu et al., 2025).

**Figure 7:**
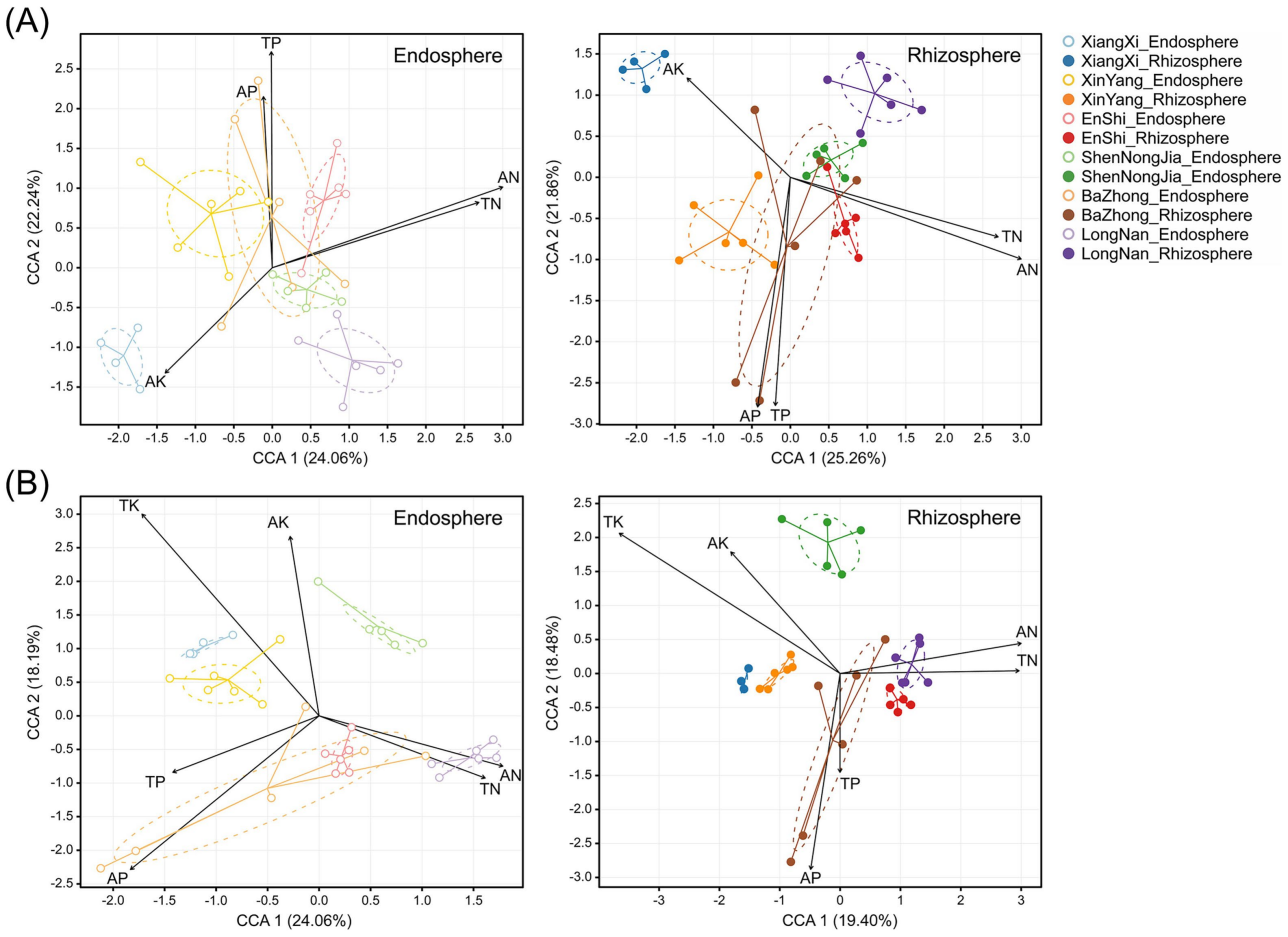
Canonical correspondence analysis (CCA) of the influences of different soil nutrients on bacterial **(A)** and fungal **(B)** communities in the root endosphere and rhizosphere of *Changnienia amoena* across habitats. Only soil nutrients having significant influences are shown. The number of samples (*n*) per habitat is as follows: LongNan (*n* = 6), XinYang (*n* = 6), EnShi (*n* = 6), ShenNongJia (*n* = 5), XiangXi (*n* = 3), and BaZhong (*n* = 6).

Next, canonical correspondence analysis (CCA) and PERMANOVA were performed to evaluate the influences of soil nutrients on root endophyte and rhizosphere microbial communities of *C. amoena*, respectively. The results showed that TN, AN, TP, AP, and AK significantly contributed to the distribution of bacterial communities (Figure 7A), and TN, AN, TP, AP, TK, and AK to fungal communities in both compartments (Figure 7B). PERMANOVA showed that TN and TP significantly explained variation in bacterial and fungal community composition in both the root endosphere and rhizosphere, whereas SOC did not show significant effect in any compartment (Table 1). Additionally, most nutrients consistently explained a larger proportion of variation in rhizosphere microbial communities than in endophytic ones, indicating that rhizosphere communities exhibit stronger overall responses to soil nutrient gradients.

**Table 1:** The permutational multivariate analysis of variance (PERMANOVA) of the influences of different soil nutrients on root endosphere and rhizosphere bacterial and fungal communities of *Changnienia amoena*.

Nutrients	Bacteria				Fungi			
	Endosphere		Rhizosphere		Endosphere		Rhizosphere	
	*R* ^2^	*P*	*R* ^2^	*P*	*R* ^2^	*P*	*R* ^2^	*P*
TN	0.061	0.003	0.074	0.001	0.051	0.002	0.067	0.001
AN	0.042	0.030	0.052	0.008	0.048	0.005	0.056	0.001
TP	0.059	0.003	0.074	0.001	0.067	0.001	0.078	0.001
AP	0.043	0.042	0.050	0.016	0.046	0.003	0.049	0.005
TK	0.036	0.095	0.035	0.107	0.039	0.022	0.043	0.011
AK	0.044	0.027	0.042	0.026	0.039	0.030	0.041	0.024
SOC	0.028	0.350	0.029	0.239	0.030	0.260	0.030	0.177

**Figure 8:**
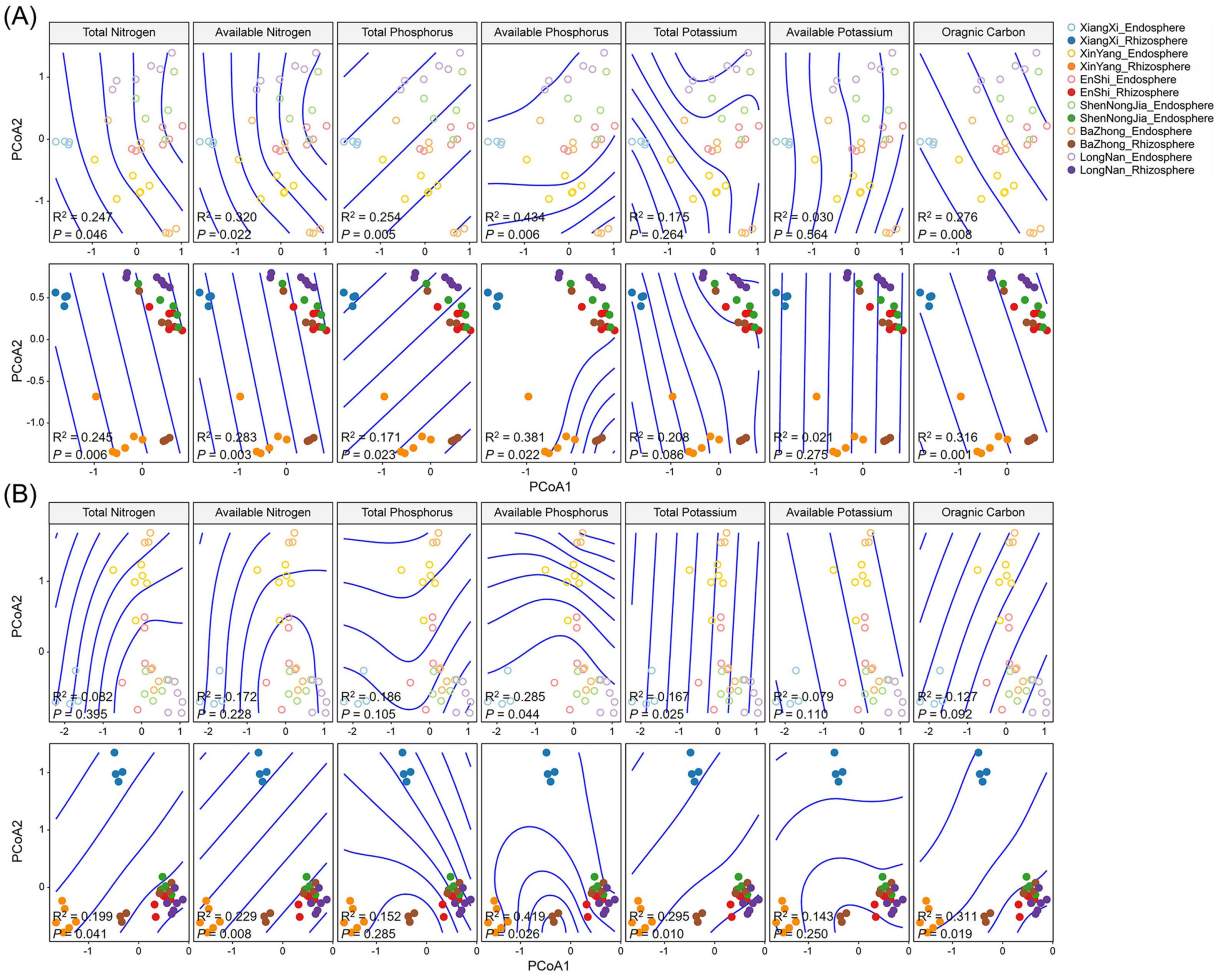
Relationships between soil nutrients and bacterial **(A)** and fungal **(B)** taxonomic composition (ordination conducted using PCoA) in the root endosphere and rhizosphere of *Changnienia amoena* across habitats. The number of samples (*n*) per habitat is as follows: LongNan (*n* = 6), XinYang (*n* = 6), EnShi (*n* = 6), ShenNongJia (*n* = 5), XiangXi (*n* = 3), and BaZhong (*n* = 6).

To gain deeper insights into the influences of soil nutrients on the root-associated microbial communities of *C. amoena*, a generalized additive model was used to characterize the relationships between individual soil nutrient and microbial taxonomic composition in the root endosphere and rhizosphere, respectively. All the tested nutrients, except TK and AK, were significantly associated with bacterial community composition in both root endosphere and rhizosphere (Figure 8A). Thus, the effects of soil nutrients on bacterial communities were consistent in both root-associated compartments. However, the effects of soil nutrients on fungal community composition differed between the root endosphere and rhizosphere. Only AP and TK were significantly associated with fungal communities in the root endosphere, whereas all tested nutrients except TP and AK showed significant effects in the rhizosphere (Figure 8B). Overall, fungal community composition in the rhizosphere was more sensitive to soil nutrient variations than that in the root endosphere, which is consistent with the PERMANOVA results (Table 1). Furthermore, linear models were used to assess the relationships between soil nutrients and microbial richness. None of the measured nutrient exhibited a significant influence on bacterial richness in either the root endosphere or the rhizosphere (Supplementary Figure S4A), whereas fungal richness in the root endosphere was significantly and positively correlated with TN, and fungal richness in the rhizosphere was significantly and positively correlated with TN, AN, and SOC (Supplementary Figure S4B). Together, soil nutrients consistently shaped bacterial community composition in both compartments, while their effects on fungal communities and richness differed between the root endosphere and rhizosphere.

**Figure 9:**
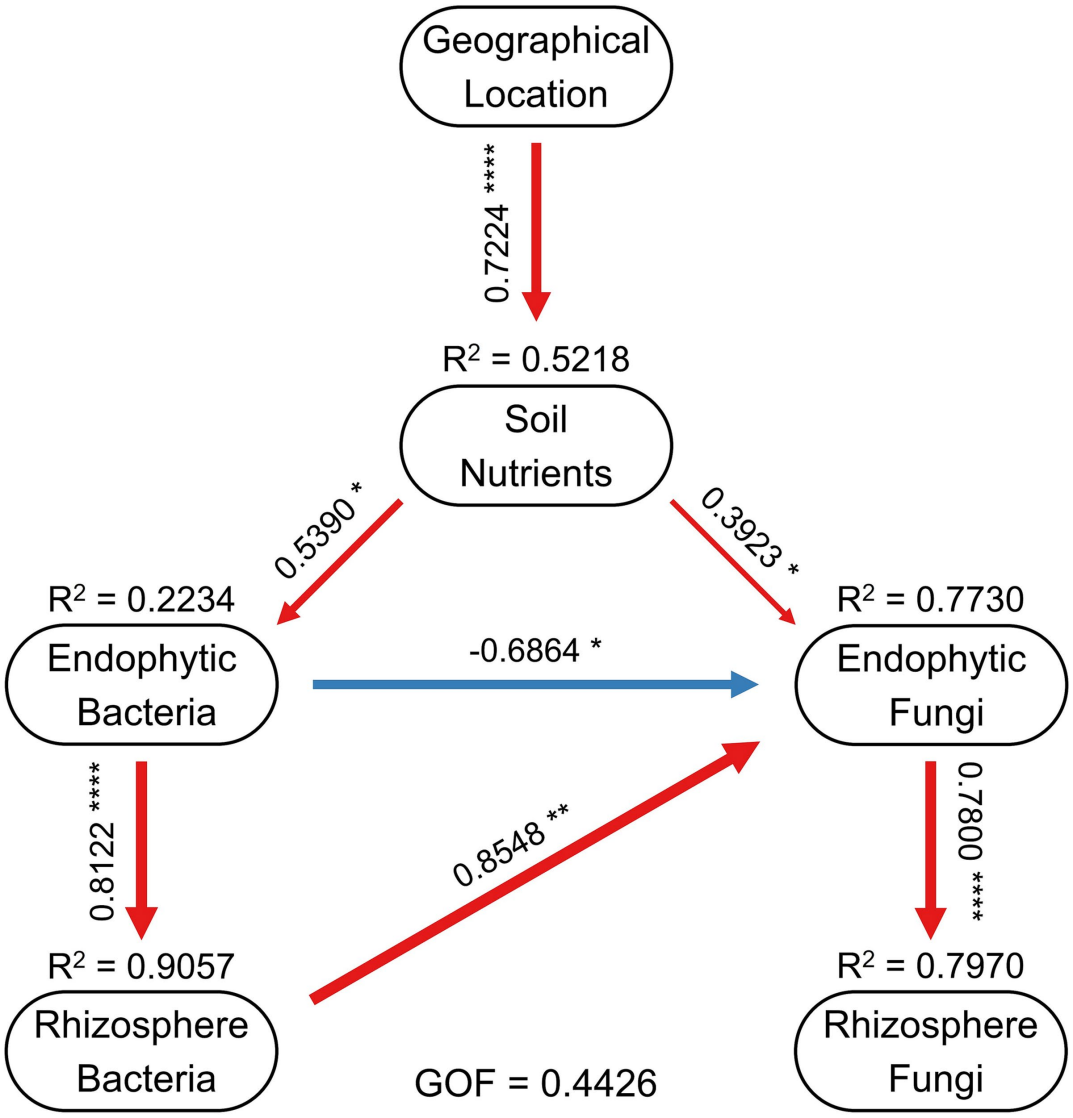
Partial least squares path model (PLS-PM) showing the relationships among geographical location, soil nutrients, and bacterial and fungal communities in the root endosphere and rhizosphere of *Changnienia amoena*. Red and blue arrows represent direct and indirect effects, respectively. Numbers along the paths represent standardized path coefficients (*p < 0.05, **p < 0.01, ****p < 0.0001). *R*^2^ values represent the proportion of variance explained for each dependent variable. GOF indicates the overall goodness-of-fit of the model. Only significant effects are shown.

Finally, a partial least squares path model (PLS-PM) was employed to elucidate the multivariate relationships among geographical location, soil nutrients and bacterial and fungal communities in the root endosphere and rhizosphere (Figure 9). Geographical location exhibited a strong positive effect on soil nutrients (*β* = 0.7224), explaining 52.18% of its variance (*R*^2^ = 0.5218). Soil nutrients, in turn, showed significant positive effects on both root endosphere bacterial (*β* = 0.5390) and fungal communities (*β* = 0.3923). Additionally, root endosphere bacterial and fungal communities were strongly linked to their corresponding rhizosphere communities, with significant positive path coefficients from endophytic to rhizosphere bacteria (*β* = 0.8122) and from endophytic to rhizosphere fungi (*β* = 0.7800). However, the direct effects of geographical location on bacterial and fungal communities in both root endosphere and rhizosphere were weak, indicating that geographical influences on microbial communities were largely mediated by soil nutrients rather than acting directly. Notably, the standardized path coefficient from rhizosphere bacterial communities to root rhizosphere fungal communities was negative (*β* = −0.6864), whereas rhizosphere bacterial communities showed a strong positive effect on root endosphere fungal communities (*β* = 0.8548). Thus, these pathways suggest that the effect of bacterial communities on fungal communities in the root endosphere of *C. amoena* is primarily indirect, mediated through rhizosphere bacterial communities. Overall, the PLS-PM indicates that soil nutrients mediate variation in root-associated bacterial and fungal communities across different native habitats.

## 4 Discussion

Orchids are highly sensitive to habitat fragmentation, which can also lead to the spatial distribution and environment-specific evolution of plant root-associated microbiomes. Root-associated microbiomes play pivotal roles in regulating plant growth and survival. Therefore, understanding the biogeographic patterns, assembly processes, and functional potential of these microbiomes can provide new insight for the conservation of endangered orchids. *C. amoena* is a rare and endangered orchid species endemic to China (Liu et al., 2025). Due to its unique taxonomic status and considerable medicinal values, it has received increasing attention in conservation biology (Liu et al., 2024). Although root-associated microbiomes are often regarded as an extension of plant genome (Li et al., 2020), the microbial communities in the root endosphere and rhizosphere of *C. amoena* remain largely uncharacterized. In this study, we provide the first comprehensive characterization of the root-associated bacterial and fungal communities of *C. amoena* across six native habitats. Our findings not only fill a critical knowledge gap regarding the microbiome of this endangered orchid but also highlight the potential roles of root-associated microbiomes in supporting orchid survival and resilience.

### 4.1 Biogeographic patterns of root-associated microbiomes

The root-associated bacterial and fungal communities of *C. amoena* varied across both locations and compartments, with locations exerting a more pronounced influence (Figures 1C,D). Moreover, similarity of both bacterial and fungal communities significantly decreased with increasing geographic distances (Figures 6A,B), demonstrating clear biogeographic patterns in the root-associated microbiomes of *C. amoena*. Similar patterns have also been reported in other plants previously. For example, bacterial communities in the root endosphere and rhizosphere of *Pinus muricata* were shaped more strongly by locations than compartments (Berrios et al., 2023), while root-associated fungal communities across several plant families were found to differ significantly across locations (Hogan et al., 2023). Notably, the PLS-PM model showed that geographical location explained a large proportion of soil nutrient variation (Figure 9). Then the soil nutrients showed significant positive effects on bacterial and fungal communities in the root endosphere, indicating that the observed microbial biogeographic patterns may result from habitat-driven differences in soil nutrient availability (Supplementary Figure S3). Previously, TN, AN, TP, TK, and AK have been reported as major drivers of rhizosphere bacterial and fungal communities of *Chromolaena odorata* (Zhang et al., 2024). Likewise, TP and AP were shown to explain major variations in root-associated fungal communities (Hogan et al., 2023). In this study, TN and TP had the strongest influences on both bacterial and fungal communities in the root endosphere and rhizosphere of *C. amoena* (Table 1). Additionally, rhizosphere microbial assemblages are more strongly structured by soil nutrients compared to endophytic communities.

In the root-associated compartments of *C. amoena*, bacterial diversity showed no significant correlation with latitude (Figure 6C), whereas fungal diversity exhibited significant negative correlations with latitude (Figure 6D). Soil is regarded as a seed bank of plant root-associated microbiota (Bai et al., 2022), and thus changes in soil microbial communities, which can be driven by nutrients, pH, temperature, moisture, or other factors (Frindte et al., 2019), may lead to changes in root-associated microbiomes. Some studies show declining soil bacterial diversity with increasing latitude, while fungal diversity follows a unimodal distribution pattern (Shi et al., 2014; Fu et al., 2023); others show a hump-shaped pattern of bacterial diversity with latitude, with fungal diversity either decreasing or showing weak latitudinal trends (Zhang et al., 2019; Liu et al., 2020). Notably, these patterns of soil microbiomes are somewhat distinct from those observed in root-associated microbiomes observed in this study, highlighting that the distribution of root-associated microbial communities is shaped not only by soil factors but also by plant–soil feedbacks (Kaur and Sharma, 2021). For example, plant root architecture and exudates can alter nutrient conditions in surrounding soils, thereby influencing rhizosphere microbial communities, which can in turn affect host plant performance (Zhao et al., 2021). Generally, the root-associated microbiomes of *C. amoena* show pronounced biogeographic patterns particularly shaped by soil nutrients with TN and TP as key factors. It should be acknowledged that, in addition to soil nutrients, other environmental factors, such as soil pH, moisture, and light availability, are also known to influence plant-microbe interactions and root-associated microbiome assembly, and thus should be considered in future studies.

### 4.2 Microbial compositions and functions in root-associated compartments

Among the top 20 most abundant bacterial genera detected in the root endosphere and rhizosphere of *C. amoena*, *Pseudomonas*, *Flavobacterium*, *Sphingomonas*, *Rhizobium*, *Bacillus*, and *Streptomyces* are commonly observed as orchid root-associated bacteria and show plant growth-promoting abilities (Kaur and Sharma, 2021). Notably, nitrogen-fixing genera such as *Bradyrhizobium*, *Rhizobium*, and *Mesorhizobium* were enriched, particularly in the roots of *C. amoena* (Figure 2A), which might be related to the enriched bacterial predicted functions related to nitrogen cycles (Figure 2B). Although these bacteria typically form root nodules in legumes, they can also promote the growth of non-legume plants by producing phytohormones, solubilizing precipitated phosphorus, mineralizing organic phosphorus, secreting siderophores, degrading ethylene, and inhibiting pathogens (Mehboob et al., 2009; Ormeño-Orrillo and Martínez-Romero, 2019). Moreover, *Bradyrhizobium* possesses the capability for photosynthesis (Avontuur et al., 2023), which is consistent with the enriched bacterial functional potential of phototrophy, photoheterotrophy, and photoautotrophy observed in root-associated compartments (Figure 2B). This trait may also contribute to the dominant distribution of *Bradyrhizobium* in forest soil (VanInsberghe et al., 2015). Since soil is regarded as a seed bank of plant root-associated microbiota (Bai et al., 2022), this ecological advantage may help to explain why *Bradyrhizobium* is the most abundant genus in the root-associated compartments of *C. amoena* (Figure 2A). However, the direct influences of *Bradyrhizobium*, *Rhizobium*, and *Mesorhizobium* on seed germination, protocorm development, seedling establishment, and then distribution of *C. amoena* deserve further exploration, which can support the function prediction in this study.

Among the top 20 most abundant fungal genera in the root endosphere and rhizosphere of *C. amoena*, *Astraeus*, *Amanita*, and *Russula* were identified as typical OMF (Figure 2C). Notably, *Astraeus* and *Amanita* represented dominant genera in the roots of *C. amoena* collected from LongNan, whereas *Russula* was most abundant in the rhizosphere of *C. amoena* from ShenNongJia. Although OMF are indispensable for orchids, the Waiting Room Hypothesis proposes that mycorrhizal partners originate from saprobic fungi through an intermediate endophytic stage (Selosse et al., 2022). In line with this hypothesis, ecological guild prediction of the fungal communities revealed an enrichment of saprobic, endophytic, and mycorrhizal trophic modes in both root endosphere and rhizosphere of *C. amoena* (Figure 2D). Among non-OMF taxa, *Mortierella* was consistently abundant across all habitats in both root-associated compartments (Figure 2C), which corroborates a previous report of its isolation from *C. amoena* roots (Jiang et al., 2011). *Mortierella* has been reported as a saprophytic fungus with multiple agricultural benefits, including enhancing gibberellic acid production *in planta* and increasing soil phosphorus availability (Li et al., 2020). Given that AP significantly influenced both bacterial and fungal communities (Figures 7, 8), *Mortierella* may affect the root-associated microbiomes of *C. amoena* by enhancing phosphorus availability. *Hygrocybe*, which has been reported neither mycorrhizal nor saprotrophic (Tello et al., 2014), was most abundant in the root endosphere of *C. amoena* from ShenNongJia. Other genera enriched in root-associated compartments were also reported to promote plant health and development, such as *Trichoderma* and *Exophiala* (Xu et al., 2020; Contreras-Cornejo et al., 2024). *Fusarium* was enriched in root-associated compartments from XiangXi, XinYang, and BaZhong, with a higher relative abundance in the root endosphere than in the rhizosphere. However, this genus is reported as both root endophytes (Shamsudin et al., 2025) and pathogens in orchids (Srivastava et al., 2018). *Fusarium* may act as conditional or opportunistic pathogens, coexisting with host plants without causing disease symptoms under non-stressful environmental conditions and in the presence of a stable root-associated microbiome (Hardoim et al., 2015). Collectively, these findings highlight the diversity of fungi in the root endosphere and rhizosphere of *C. amoena*, including several groups with plant growth-promoting potentials, which may contribute to the health, adaptation, and distribution of this endangered orchid.

### 4.3 Multipartite interactions among plants, mycorrhizal fungi and bacteria

In the microbial co-occurrence networks across habitats, several ASVs were identified as mycorrhizal fungi (Figure 4). Among these, Ceratobasidiaceae, Tulasnellaceae, and Serendipitaceae are collectively referred to as rhizoctonias and are known to establish mycorrhizal associations exclusively with orchids (Selosse et al., 2022). Additionally, ectomycorrhizal fungi, mainly from Sebacinaceae and Thelephoraceae, are frequently detected in orchid roots as well. These mycorrhizal fungal ASVs exhibited significant positive relationships with many bacterial ASVs (Figure 4), indicating intimate interactions among *C. amoena*, mycorrhizal fungi, and bacteria. For example, MHBs can enhance mycorrhizal colonization by stimulating spore germination and mycelial expansion, as well as increasing root surface area to facilitate fungal colonization. Consequently, MHBs can double the colonization rate of mycorrhizal fungi and promote plant growth (Berrios et al., 2023). In the root-associated microbial networks of *C. amoena*, *Bradyrhizobium*, *Rhizobium*, *Mesorhizobium*, and *Nitrospira* showed significant positive correlations with mycorrhizal fungal ASVs (Figure 4). *Bradyrhizobium* has been reported to positively associate with mycorrhizal fungal abundance and identified as a strong predictor of mycorrhizal colonization in pine forests (Berrios et al., 2023). A similar trend was observed in the root-associated compartments of *C. amoena*. In detail, *Bradyrhizobium* was more abundant in BaZhong and XiangXi compared to other habitats (Figure 2A), which exhibited more complex microbial co-occurrence networks with higher numbers of mycorrhizal fungal ASVs (Figure 4). Additionally, mycorrhization can also be stimulated by *Pseudomonas*, *Streptomyces*, *Rhizobium*, and *Bacillus* (Frey-Klett et al., 2007), which were also abundant in the root-associated compartments of *C. amoena* (Figure 2A). Importantly, the multipartite interactions among plants, mycorrhizal fungi and bacteria can support plant growth and productivity (Kaur and Sharma, 2021) by enhancing nutrient mobilization from soil minerals, fixing atmospheric nitrogen, and protecting plants against root pathogens (Frey-Klett et al., 2007). Notably, although the soil nutrient levels in XiangXi and BaZhong are relatively lower than those in other habitats (Supplementary Figure S3), these two locations exhibit the most complex microbial networks (Figure 4). Therefore, the *C. amoena* individuals in XiangXi and BaZhong may benefit from their root-associated microbiomes. Overall, our results highlight close associations among *C. amoena*, mycorrhizal fungi, and root-associated bacteria, which may be relevant to plant performance and persistence across its native habitats. Therefore, the simulation of microbial interactions of native habitats may provide an efficient strategy for the conservation of endangered plants.

### 4.4 Implications of root-associated microbiomes for the conservation of *Changnienia amoena*

Our results demonstrate that the persistence of *C. amoena* in its native habitats is closely associated with the composition, functional potential, and interaction structure of its root-associated microbiomes, highlighting the importance of integrating microbial ecology into conservation strategies for endangered orchids. The widespread occurrence of OMF across root-associated compartments and habitats (Figures 2C,D) indicates that these fungal taxa are essential for the long-term survival of *C. amoena*, particularly given the obligate dependence of orchids on mycorrhizal symbiosis for seed germination and early development (McCormick et al., 2018). Additionally, the root-associated fungal communities can be affected by soil nutrients (Figures 7B, 8B), suggesting their vulnerability to habitat disturbance. Therefore, effective *in situ* conservation should prioritize the protection of undisturbed native habitats and intact mycorrhizal networks that support plant population persistence and regeneration. In parallel, *ex situ* conservation of *C. amoena* may benefit from inoculation with locally adapted OMF to enhance seed germination, seedling establishment, as previously demonstrated in other orchids (Yang et al., 2024).

In addition to fungi, the enrichment nitrogen-cycling bacterial genera such as *Bradyrhizobium*, *Rhizobium*, and *Mesorhizobium* (Figures 2A,B) suggests that root-associated bacterial partners may contribute to nitrogen acquisition of *C. amoena* and partially compensate for nitrogen limitation in native habitats (Trivedi et al., 2020). Notably, these nitrogen-cycling bacterial genera were identified as core microbiome members associated with *C. amoena* across native habitats (Figure 3A). Therefore, soil disturbance and excessive fertilization should be minimized in native populations of *C. amoena*, as maintaining microbiome-mediated nutrient cycling processes is critical for sustaining ecosystem resilience.

Further, the strong positive associations between OMF and beneficial bacterial taxa revealed by microbial co-occurrence network analyses (Figure 4) indicate that *C. amoena* relies on multipartite plant-fungus-bacterium interactions rather than single microbial partners. More importantly, geographically distinct populations of *C. amoena* are supported by habitat-specific microbiomes rather than a uniform microbial assemblage (Figures 1C,D). Collectively, the conservation of this endangered orchid should not focus solely on the plant itself but also consider the preservation of its associated microbiome.

## 5 Conclusion

In summary, understanding the biogeographic patterns, assembly processes, and functional potential of root-associated microbiomes of endangered orchids is critical for their conservation. Here, we present the first comprehensive characterization of the root-associated microbiomes of *C. amoena* across six native habitats within its natural range. Several fungal and bacterial taxa with reported beneficial roles for plant growth and development were identified, including OMF. Additionally, distinct microbial biogeographic patterns were strongly shaped by soil nutrients, particularly TN and TP. Collectively, these findings not only expand the understanding of the plant–soil-microbe interactions in endangered orchids but also provide a scientific basis for integrating microbial ecology into orchid conservation and for developing effective management strategies. Future studies incorporating bulk soil microbiomes will further disentangle soil microbial reservoirs, host selection processes, and compartment-specific enrichment patterns of root-associated microbiomes.

## Data Availability

The 16S rRNA and ITS amplicon sequencing datasets of root-associated bacteria and fungi of Changnienia amoena are available in the NCBI BioProject database under accession numbers PRJNA1322054 (https://www.ncbi.nlm.nih.gov/bioproject/?term=PRJNA1322054) and PRJNA1321960 (https://www.ncbi.nlm.nih.gov/bioproject/?term=PRJNA1321960).
