# Supplementary material for: Biogeographic patterns, assembly processes, and functional potential of root-associated microbiomes across the native range of the endangered orchid Changnienia amoena
Source: Front Microbiol. 2026 Feb 10;17:1752368. doi: 10.3389/fmicb.2026.1752368 (PMC12929374; doi:10.3389/fmicb.2026.1752368)
Supplement: Supplementary file 2 [file Image_1.pdf]

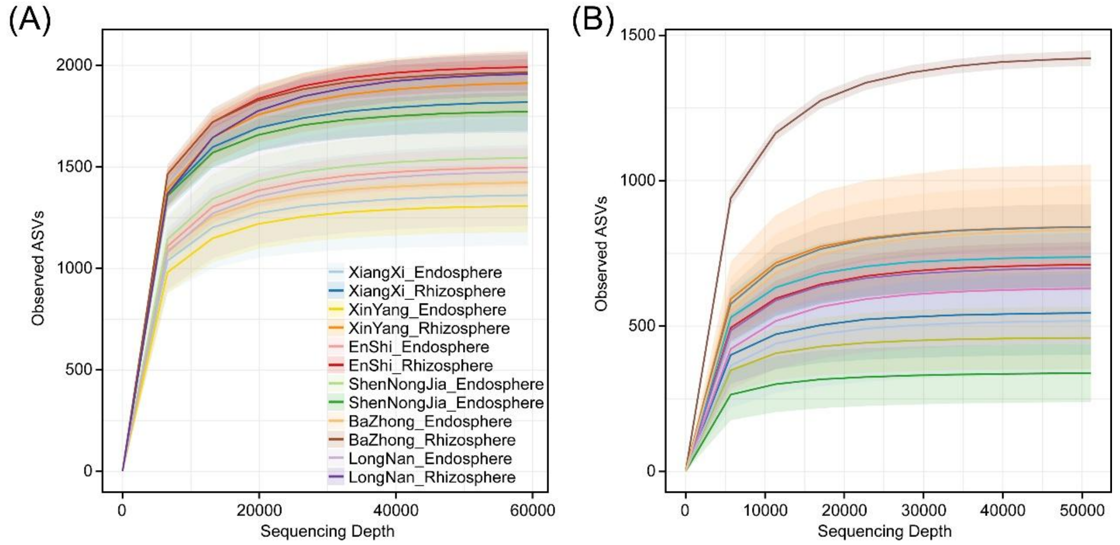

Supplementary Figure S1 Rarefaction curves generated according to bacterial **(A)** and fungal **(B)** richness (the average observed ASV number in each group) with the sequencing depth.

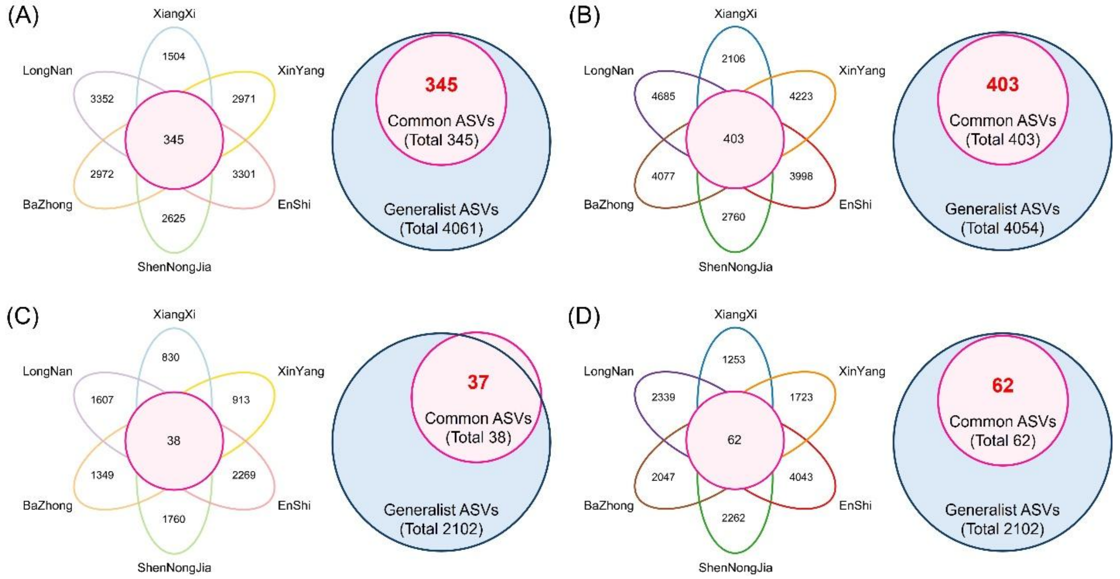

Supplementary Figure S2 Core bacterial **(A and B)** and fungal **(C and D)** microbes in the root endosphere (A and C) and rhizosphere (B and D) of *Changnienia amoena* across habitats. Venn diagrams show the number of Amplicon Sequence Variants (ASVs) unique to each habitat and those shared among all habitats. ASVs that were shared across habitats and classified as generalists were defined as core microbes.

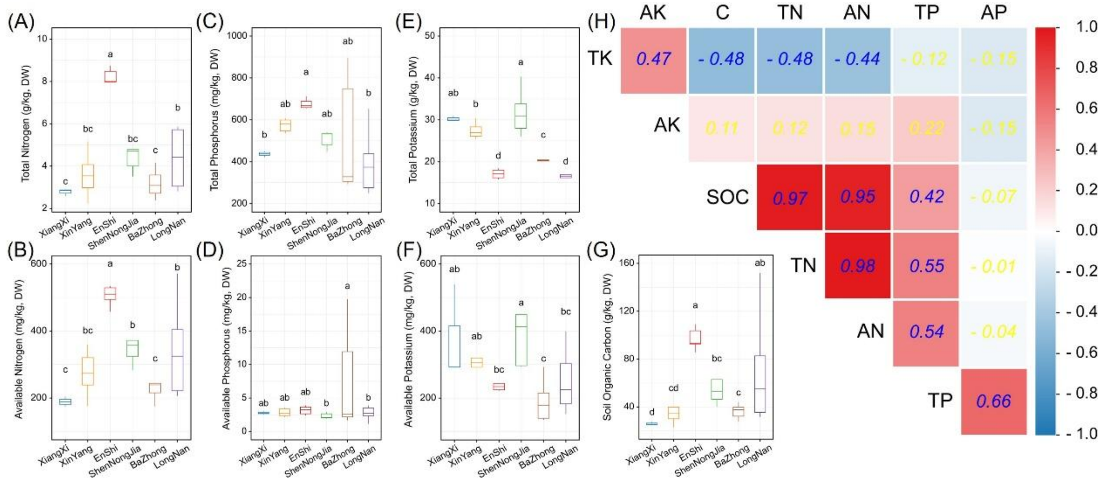

Supplementary Figure S3 Total nitrogen **(A)**, available nitrogen **(B)**, total phosphorus **(C)**, available phosphorus **(D)**, total potassium **(E)**, available potassium **(F)**, organic carbon **(G)** content in the soil collected from different native habitats and their correlations between each other. Horizontal lines within boxes indicate medians. Tops and bottoms of boxes indicate 75th and 25th quartiles, respectively. Upper and lower whiskers extend 1.5× the interquartile range from the upper edge and lower edge of the box, respectively. Different letters indicate significant differences among different locations (P < 0.05) based on one-way ANOVA. Pearson correlation coefficients were shown in the heatmap. The number of samples (n) per habitat is as follows: LongNan (n = 6), XinYang (n = 6), EnShi (n = 6), ShenNongJia (n = 5), XiangXi (n = 3), and BaZhong (n = 6).

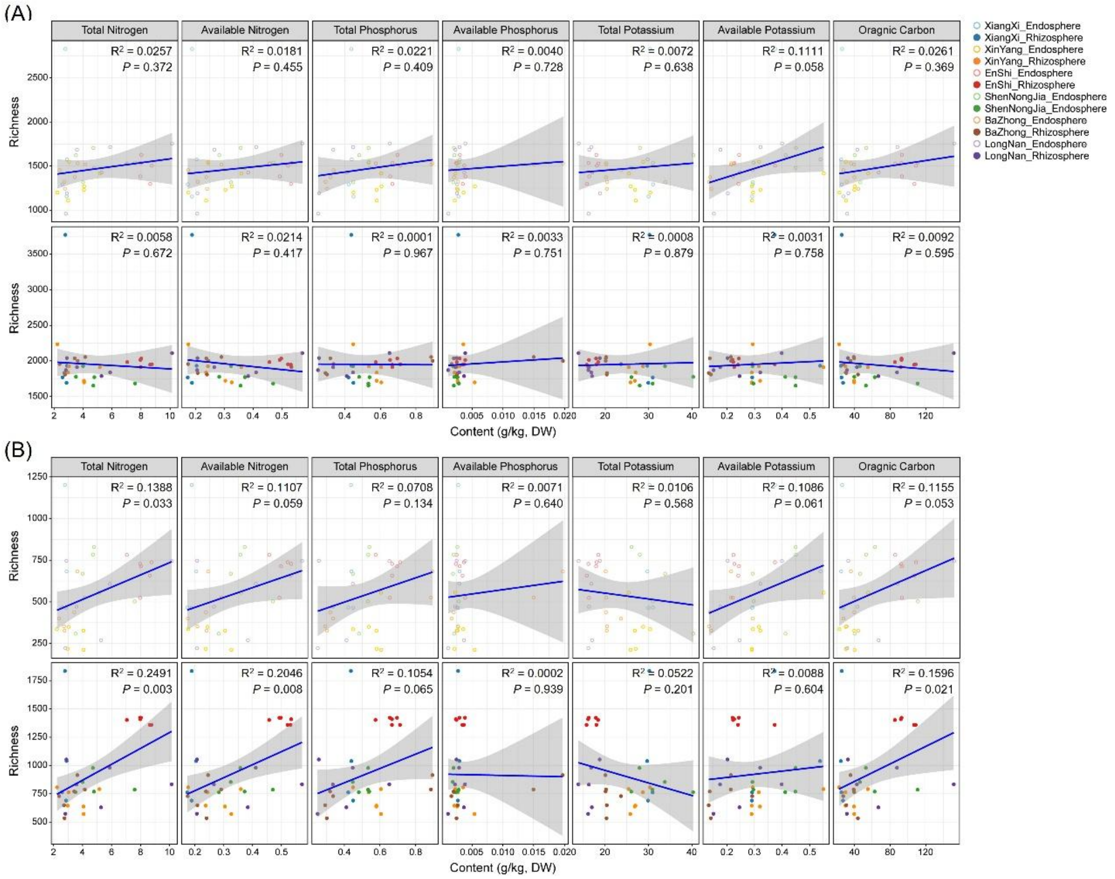

Supplementary Figure S4 Correlations between soil nutrients and bacterial **(A)** and fungal **(B)** community richness in the root endosphere and rhizosphere of *Changnienia amoena*. The number of samples (n) per habitat is as follows: LongNan (n = 6), XinYang (n = 6), EnShi (n = 6), ShenNongJia (n = 5), XiangXi (n = 3), and BaZhong (n = 6).
